# Chemical Reactivity of Supported ZnO Clusters: Undercoordinated Zinc and Oxygen Atoms as Active Sites

**DOI:** 10.1002/cphc.202000747

**Published:** 2020-11-13

**Authors:** Xiaojuan Yu, Jannik P. Roth, Junjun Wang, Eric Sauter, Alexei Nefedov, Stefan Heißler, Gianfranco Pacchioni, Yuemin Wang, Christof Wöll

**Affiliations:** ^1^ Institute of Functional Interfaces Karlsruhe Institute of Technology Eggenstein-Leopoldshafen 76344 Germany; ^2^ Dipartimento di Scienza dei Materiali Università Milano-Bicocca Via R. Cozzi 55 20125 Milano Italy

**Keywords:** active sites, density functional calculations, infrared reflection absorption spectroscopy, surface chemistry, thin films, ZnO

## Abstract

The growth of ZnO clusters supported by ZnO‐bilayers on Ag(111) and the interaction of these oxide nanostructures with water have been studied by a multi‐technique approach combining temperature‐dependent infrared reflection absorption spectroscopy (IRRAS), grazing‐emission X‐ray photoelectron spectroscopy, and density functional theory calculations. Our results reveal that the ZnO bilayers exhibiting graphite‐like structure are chemically inactive for water dissociation, whereas small ZnO clusters formed on top of these well‐defined, yet chemically passive supports show extremely high reactivity ‐ water is dissociated without an apparent activation barrier. Systematic isotopic substitution experiments using H_2_^16^O/D_2_^16^O/D_2_^18^O allow identification of various types of acidic hydroxyl groups. We demonstrate that a reliable characterization of these OH‐species is possible via co‐adsorption of CO, which leads to a red shift of the OD frequency due to the weak interaction via hydrogen bonding. The theoretical results provide atomic‐level insight into the surface structure and chemical activity of the supported ZnO clusters and allow identification of the presence of under‐coordinated Zn and O atoms at the edges and corners of the ZnO clusters as the active sites for H_2_O dissociation.

## Graphical abstract

**Chemical nature of small ZnO clusters**: The combined infrared reflection absorption spectroscopy (IRRAS) and theoretical results reveal that the Ag(111)‐supported graphitic ZnO bilayers are chemically inactive. Significant activity is observed when small ZnO clusters are supported by these bilayers. This apparently barrier‐free water dissociation takes place at under‐coordinated Zn and O atoms at the edges and corners of the ZnO nanostructures. Various acidic hydroxyl species are identified by isotopic substitution experiments and the formation of weak H bonds to coadsorbed CO molecules.

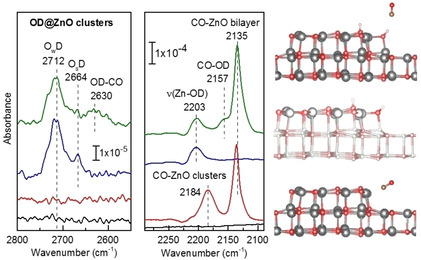

## 1 Introduction

The catalytic activity of oxide nanoparticles (NPs) is a topic of pronounced interest. Although in many cases real catalysts consist of metal particles supported by such oxide NPs, also the oxides alone exhibit chemical activity.[^1^, ^2^, ^3^, ^4^] However, the experimental characterization of powdery materials represents a major problem since such samples typically contain particles of different sizes and shapes exhibiting various orientations and possibly also defects of different types. For this reason, investigations of oxide nanoparticles with well‐defined structures are an important goal, in particular with regard to the validation of theoretical results.

The surface chemistry of water on metal oxide substrates is a central issue in numerous research areas including catalysis and photocatalysis.[^5^, ^6^, ^7^, ^8^] Especially the role of water on zinc oxide (ZnO) has drawn great attention, because it is involved in a variety of important reactions, such as methanol synthesis via syngas conversion and hydrogen production by the water gas shift reaction as well as the splitting of water.[^9^, ^10^, ^11^, ^12^, ^13^, ^14^, ^15^, ^16^]

Metal‐supported ZnO thin films are known to feature tunable structural and chemical properties which differ substantially from those of the bulk wurtzite ZnO.[^17^, ^18^, ^19^, ^20^, ^21^, ^22^, ^23^, ^24^, ^25^, ^26^, ^27^, ^28^] To date, experimental and theoretical works have focused predominantly on well‐defined two‐dimensional ZnO films, many of them of bilayer thickness, while much less information is available about small ZnO clusters (or islands) supported on such ZnO bilayers.

As regards the ZnO bilayers supported on Cu(111) and Ag(111) single‐crystal surfaces, our experimental results acquired by infrared reflection absorption spectroscopy (IRRAS) using CO as a probe molecule have revealed that the surface chemistry of ZnO bilayers is quite different from that of ZnO single crystals surfaces.[^17^, ^28^] This conclusion was directly confirmed by the fact that the stretch frequencies of CO bound to these bilayers amount to 2116 cm^−1^ for Cu‐ZnO^[17]^ and 2138 cm^−1^ for Ag‐ZnO,^[28]^ which are substantially different from the frequencies observed for CO adsorbed on bulk ZnO in the form of single crystals or powders.[^29^, ^30^, ^31^] On the basis of theoretical work these observations were explained by a different structure of the ZnO bilayers, instead of the bulk wurtzite‐type arrangement the presence of graphitic adlayers as discussed in previous works was proposed.[^27^, ^28^]

In the present paper we describe the growth of ZnO thin films on Ag(111) substrates. After preparation, the interaction of these 2D oxide systems with water were thoroughly investigated, using temperature‐dependent IRRAS and grazing‐emission X‐ray photoelectron spectroscopy (XPS) in conjunction with density functional theory (DFT) calculations. The temperature‐resolved IRRAS approach not only allows for gaining information about desorption of adsorbed species, but also enables to monitor thermally induced reactions. For example, recent results for water on wurtzite‐type ZnO(10$\bar{1}$ 0) surfaces revealed the thermal diffusion of water monomers forming dimer species and the subsequent dissociation of water dimers via proton transfer.^[32]^ In this work, we find that the ZnO bilayers are chemically rather inactive and show associative adsorption of water. Only after the presence of small ZnO clusters on these bilayers the formation of hydroxyl species is observed. The ZnO clusters exhibit significantly enhanced chemical activity, leading to an apparently non‐activated dissociative adsorption of water. The assignment of various acidic hydroxyl groups obtained by the dissociation process is supported by isotopic substitution experiments (H_2_^16^O/D_2_^16^O/D_2_^18^O). Vibrational shifts caused by interaction with coadsorbed CO are also analyzed. These results are rather different from those of water adsorption on wurtzite‐type ZnO single crystal surfaces.[^32^, ^33^, ^34^, ^35^, ^36^] A careful theoretical analysis allows rationalizing the experimental findings and provides a rather consistent description of the chemical activity of small ZnO clusters.

## 2 Results and Discussion

### 2.1 Adsorption of D_2_O on Graphite‐Like ZnO Bilayers

The well‐defined graphitic ZnO bilayer was formed by oxidation of the AgZn(111) single‐crystal surface in O_2_ atmosphere (1×10^−5^ mbar) at 600 K for 20 min (for details see Ref. [28]). Briefly, the oxidation of the AgZn(111) alloy substrate (Ag/Zn ratio 9 : 1) at different temperatures for different periods of time allowed for a well‐controlled growth of ZnO adlayers. The presence of a closed, planar ZnO bilayer was confirmed by the IRRAS observation of one single, sharp CO band at 2138 cm^−1^ while the Ag‐bonded CO vibration at 2121 cm^−1^ completely disappeared (see Figure S1). This conclusion was further supported by the thickness analysis of ZnO (3.4 Å) based on XPS measurements and by a thorough theoretical study using DFT calculations.^[28]^ We note that this AgZn(111) alloy oxidation approach is rather different from other methods (e. g. Zn deposition in the presence of O_2_) that typically lead to the formation of not‐closed ZnO adlayers.

**Figure 1:**
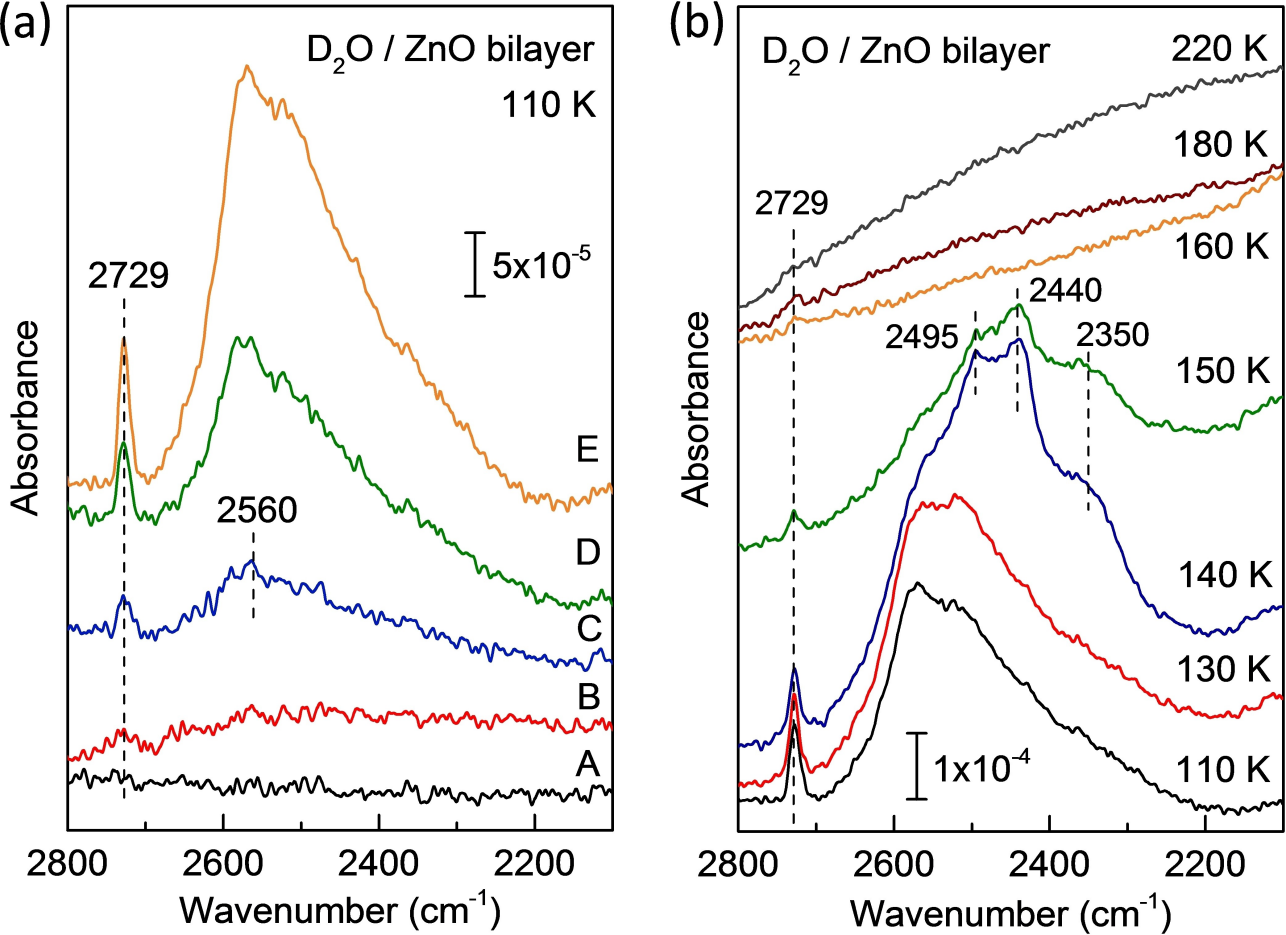
(a) IRRA spectra recorded after exposing graphite‐like ZnO bilayer to different doses of D_2_O at 110 K. (A) Clean surface and (B–E) exposure to D_2_O: (B) 0.1 L, (C) 0.2 L, (D) 0.5 L, (E) 1 L. All spectra were recorded with non‐polarized light. (b) Temperature‐dependent IRRA spectra recorded after exposing ultrathin ZnO bilayer to 1 L of D_2_O at 110 K and then heating gradually to indicated temperatures.

Figure 1 shows the IRRAS data recorded after exposing Ag(111)‐supported ZnO bilayer to various amounts of D_2_O at 110 K. After exposure up to 0.2 L of D_2_O at 110 K, a weak signal at 2729 cm^−1^ with a rather broad feature between 2700 and 2200 cm^−1^ (centered at ∼2560 cm^−1^) is observed. They are characteristic for a dangling ν(OD) band in intact water molecules and H‐bonded OD vibrations, respectively. Further exposure to D_2_O leads to a significant increase of the IR bands in intensity without saturation. These findings indicate that the ZnO surface is dominated by the chemisorbed water in the monolayer region after low dose adsorption of D_2_O (up to 0.2 L) at 110 K, whereas further exposure leads to the formation of multilayer water molecules with H‐bonded 3‐dimentional structures, as observed for water adsorption on ZnO(10$\bar{1}$ 0) single‐crystal surfaces.^[32]^

Figure 1b displays temperature‐dependent IRRAS data recorded after the formation of D_2_O multilayers on ZnO bilayers. Upon annealing to 140 K substantial changes of vibrational features are seen, and three new bands appear at 2495, 2440 and 2350 cm^−1^, revealing a structure conversion from amorphous to well‐ordered hexagonal ice.^[33]^ The crystalline ice structure is stabilized by strong intermolecular H‐bonding. After heating up to 160 K, complete desorption of multilayer D_2_O molecules occurs (see Figure 1b). In this case, the ZnO surface is covered only by chemisorbed 2‐dimentional D_2_O monolayer, as confirmed by the typical 2729 cm^−1^ band originating from the dangling OD groups of adsorbed D_2_O. Interesting, the H‐bonded OD vibrations observed for the water monolayer at 110 K (see Figure 1a), are not detectable any more. This could be explained by the formation of ordered superstructures of chemisorbed water with H‐bonded OD groups in a flat lying geometry, as discussed below in detail. We note that an ordered structure of the water adlayer cannot be formed at 110 K due to the low mobility of water molecules.[^25^, ^32^] The monolayer D_2_O molecules desorb completely after further annealing to 220 K (see Figure 1b).

Importantly, the present IRRAS data did not show any indications for OD bands at ∼2710 cm^−1^ (Figure 1), which are typical for hydroxyl species resulting from water dissociation. This finding suggests that unlike the macroscopic ZnO(10$\bar{1}$ 0) surface,^[32]^ the graphitic ZnO bilayer formed on Ag(111) is virtually inactive for water dissociation.

**Figure 2:**
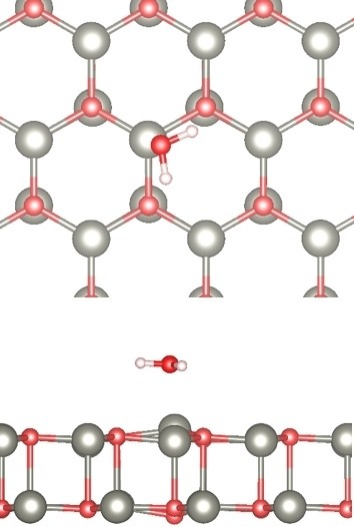
DFT‐optimized structure of H_2_O monomer adsorbed on the ZnO bilayer. Top: top view, bottom: side view. Color code: O (red), Zn (gray), H (white).

Using DFT, we modeled the adsorption of a water monomer on the unsupported ZnO bilayer. Optimizing the free‐standing bilayer (with lattice parameters fixed to those of the Ag support) in the hexagonal boron nitride structure leads to a completely flat bilayer (Figure S2). This agrees with previous theoretical results.^[27]^ In the most stable adsorption mode, the water molecule is lying flat (see Figure 2). The adsorption energy is −0.42 eV. A vibrational analysis showed three modes at 2616.1 (−58.4), 1150.5 (−7.6) and 2739.5 cm^−1^ (−63.6 cm^−1^), where the frequency shifts (in parenthesis) are given with respect to the gas phase molecule. Including the metal support does not result in significant changes. On ZnO/Ag(111) the adsorption energy becomes −0.43 eV and the computed frequencies are found at 2626.9 (−47.7), 1152.7 (−5.4) and 2750.5 cm^−1^ (−53.0 cm^−1^). The effect of the metal support on the chemical properties of the supported bilayer is negligible. Due to the flat lying adsorption mode, the vibrational contributions perpendicular to the surface are negligible and the vibrations of an isolated H_2_O molecule (symmetric and asymmetric stretching, bending) should not be detectable experimentally due to the so‐called surface selection rule.^[37]^

We also considered water dimers and we found two stable structures (see Table 1 and Figure S3). The adsorption energy/molecule for both dimer structures is considerably larger than that for the monomer. Both isomers have two vibrational modes with an important component perpendicular to the surface. Of course, it is expected that other dimer structures may exist with similar adsorption energies.

**Table 1:** Computed adsorption energy per molecule and vibrational modes for the water dimer.

	Isomer 1	Isomer 2
E_ads_/molecule [eV]	−0.57	−0.64
ν_1_ [cm^−1^]	2747.7 (−55.3)	2754.4 (−48.6)
ν_2_ [cm^−1^]	2730.9 (−72.1)	2737.4 (−65.7)

**Figure 3:**
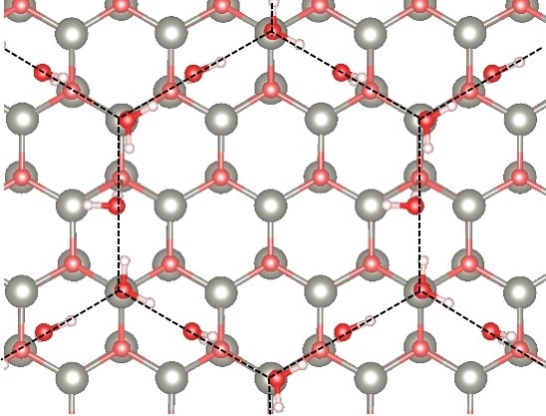
One of the possible superstructures of adsorbed water. The water molecules form a regular hexagonal pattern as indicated by the dashed lines. Color code: O (red), Zn (gray), H (white).

Ordered regular patterns of adsorbed water molecules have been studied by Deng *et al*. for the case of ZnO/Au(111).^[25]^ We investigated one of the many possible water superstructures with 3x3 periodicity (Figure 3). The adsorption energy is −0.70 eV per water molecule, slightly larger than that for the dimer structures. A vibrational analysis showed two modes at 2741.2 (−61.9) and 2715.0 cm^−1^ (−88.1 cm^−1^) as well as a multitude of modes from 2150 up to 2650 cm^−1^ typical for hydrogen bonds. The absolute values of these frequencies should be taken with care since hydrogen bonds often show a strong anharmonicity, which is not included in our approach.

The experimental results suggest that water does only adsorb molecularly on the intact ZnO bilayer. To verify this, we modeled dissociative adsorption on the unsupported ZnO bilayer by placing an OD and a D fragments on top of a Zn and O atoms of the surface, respectively. The calculations showed no spin polarization and the formation of OD^‐^ and H^+^ fragments (heterolytic dissociation). If the two fragments are placed on neighboring O and Zn surface atoms, the optimization leads to a spontaneous recombination with formation of an intact water molecule.

Placing the D and OD fragments further apart (distance ∼4 Å), the adsorption becomes metastable with an adsorption energy of +0.10 eV. In this structure, the OD fragment binds in a bridge position to two Zn atoms of the surface. A DFT vibrational analysis showed two modes at 2732.4 (−70.6) and 2689.1 cm^−1^ (−113.9 cm^−1^). These results clearly show that dissociative adsorption is unfavorable on the ZnO bilayer, in line with the experimental observations.

**Figure 4:**
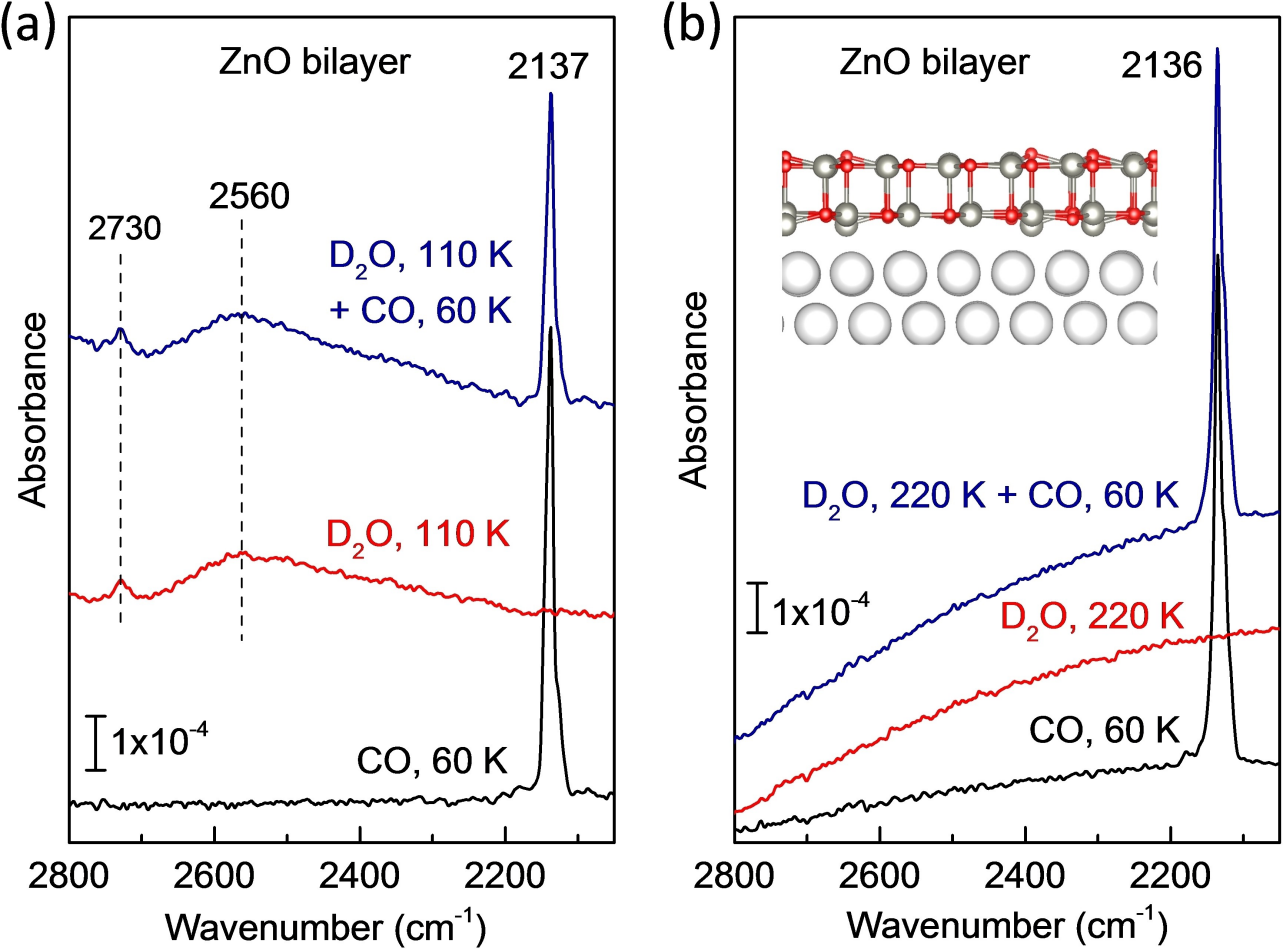
IRRA spectra obtained by exposing the ultrathin ZnO bilayer to 1 L CO at 60 K, 0.2 L D_2_O at (a) 110 K, (b) 220 K and first 0.2 L D_2_O and then 1 L CO at 60 K, respectively.

The intact adsorption of water on the graphite‐like ZnO bilayer is further supported by co‐adsorption experiments with CO. For the ultrathin ZnO bilayer, after an exposure of 1 L CO at 60 K, only one CO band at 2137 cm^−1^ is detected in IRRAS (Figure 4), indicating the presence of a uniform, defect‐free ZnO bilayer providing only one type of CO binding sites. After heating to 110 K, CO is totally desorbed, as expected from the rather low binding energy (0.24 eV).^[28]^ The spectra recorded after exposure of monolayer D_2_O (0.2 L) at 110 K show two IR bands at 2730 and 2560 cm^−1^ (broad feature), which are characteristic for H‐bonded D_2_O molecules. After subsequent re‐adsorption of 1 L CO at 60 K, the CO‐related band at 2136 cm^−1^ can still be observed, but with much lower intensity (Figure 4a). However, the amount of CO adsorption remains unchanged when the sample was subjected to exposing D_2_O at 220 K (Figure 4b), demonstrating again the inactive properties of the planar ZnO bilayer structure for water dissociation.

The DFT modeling of CO adsorption has been done for the unsupported bilayer since we have seen that the effect of the Ag(111) support on the oxide thin film is small, if any. CO binds to the zinc atoms exposed at the oxide bilayer surface with d_Zn‐C_=2.54 Å (Figure S4) and an adsorption energy of −0.21 eV (coverage of θ=^1^/_9_). The CO vibrational frequency is 2134.5 cm^−1^ with a shift of +9.0 cm^−1^ compared to the gas phase. In our previous work,^[28]^ we have found a coverage‐induced frequency shift for the CO stretching vibration, ranging from 2138 cm^−1^ (−5 cm^−1^) at high coverages to 2146 cm^−1^ (+3 cm^−1^) at low coverages. This is attributed to the lateral adsorbate‐adsorbate interactions.^[31]^ The computed frequency for adsorbed CO (the distance between two CO molecules is 9.9 Å) is in good agreement with the IR observation.

### 2.2 Adsorption of D_2_O on ZnO Clusters Supported by ZnO Bilayers

The thicker films were prepared via prolonged oxidation of AgZn(111) at 600 K for 40 min, yielding an average thickness of 4.4 Å, only slightly higher than that (3.4 Å) of a flat ZnO bilayer (for details see Ref. [28]). Our IRRAS investigations using CO as a probe molecule revealed the subsequent growth of small ZnO clusters on the well‐ordered ZnO bilayer surface (Figure S1). While the latter structure showed a sharp CO band at 2138 cm^−1^, the supported ZnO clusters was characterized by a weak and broad CO vibration at much higher frequency (2184 cm^−1^).^[28]^ It should be emphasized that the 2184 cm^−1^ feature was observed only for thickness of ZnO above 2 monolayers. This finding provided spectroscopic evidence for the formation of ZnO clusters on the closed, planar ZnO bilayer surface.

**Figure 5:**
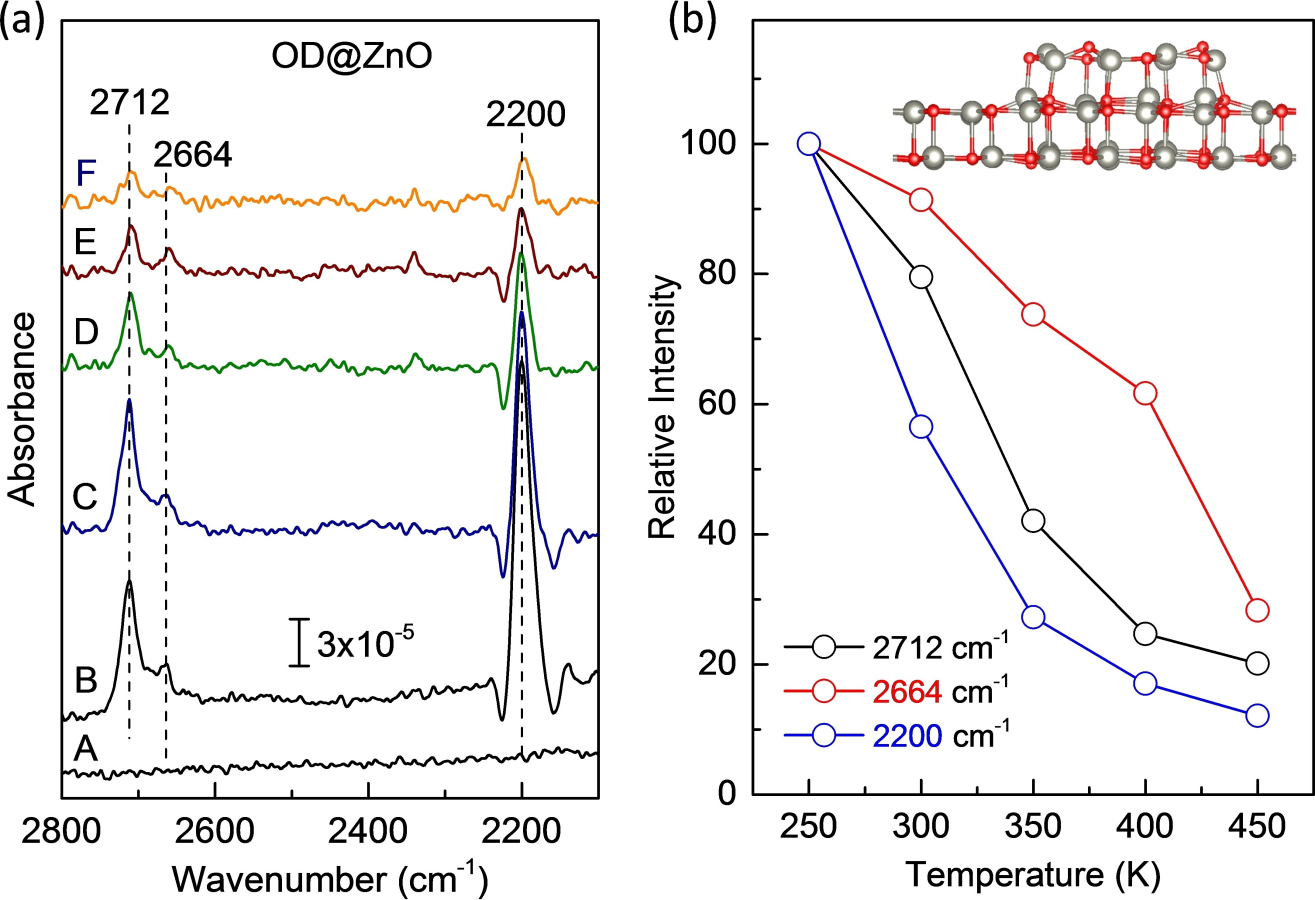
(a) Temperature‐resolved IRRAS data recorded after exposing (A) ZnO bilayer‐supported ZnO clusters to 1 L of D_2_O at (B) 250 K and heating gradually to (C) 300 K, (D) 350 K, (E) 400 K, (F) 450 K. (b) Relative intensity of different peaks as a function of sample temperature.

When small ZnO clusters are formed on the graphitic ZnO bilayer, the interaction with water changes drastically. As shown in Figure 5a, after an exposure of 1 L D_2_O at 250 K, the spectrum exhibits two high‐frequency vibrational bands at 2712 and 2664 cm^−1^. They are assigned to stretching vibrations of hydroxyl species resulting from water dissociation. The IRRAS thermal desorption spectra (Figure 5a) provide further evidence for the formation of two surface hydroxyl groups via dissociative adsorption of water. Both OD bands exhibit a rather high thermal stability and are visible even at 450 K (see Figure 5b).

Interestingly, after D_2_O adsorption a weak band appears at 2200 cm^−1^ (Figure 5a) and remains unchanged in frequency when exposed to H_2_^16^O or D_2_^18^O (not shown here), demonstrating that this band is not related to hydroxyl vibrations. We assign this band to the excitation of multiple Fuchs‐Kliewer phonons (lattice Zn−O vibrations).^[38]^ In a previous HREELS study, the fundamental surface phonon mode for wurtzite ZnO crystals was observed at ∼555 cm^−1^, with the corresponding overtones located at about 1110, 1665 and 2220 cm^−1^.[^39^, ^40^] This assignment also explains the unusual peak profile, with negative wings on the left and on the right side. The observed slight red‐shift to 2200 cm^−1^ relative to the EELS data can be explained in terms of the change of the surface phonons of the ZnO clusters (NOT the ZnO bilayers) due to the dissociative adsorption of D_2_O forming Zn‐OD species. It should be noted that the surface phonons of the flat, graphitic ZnO bilayers and the small ZnO clusters formed on them should be different from those of wurtzite ZnO crystals due to the structure modifications.

**Figure 6:**
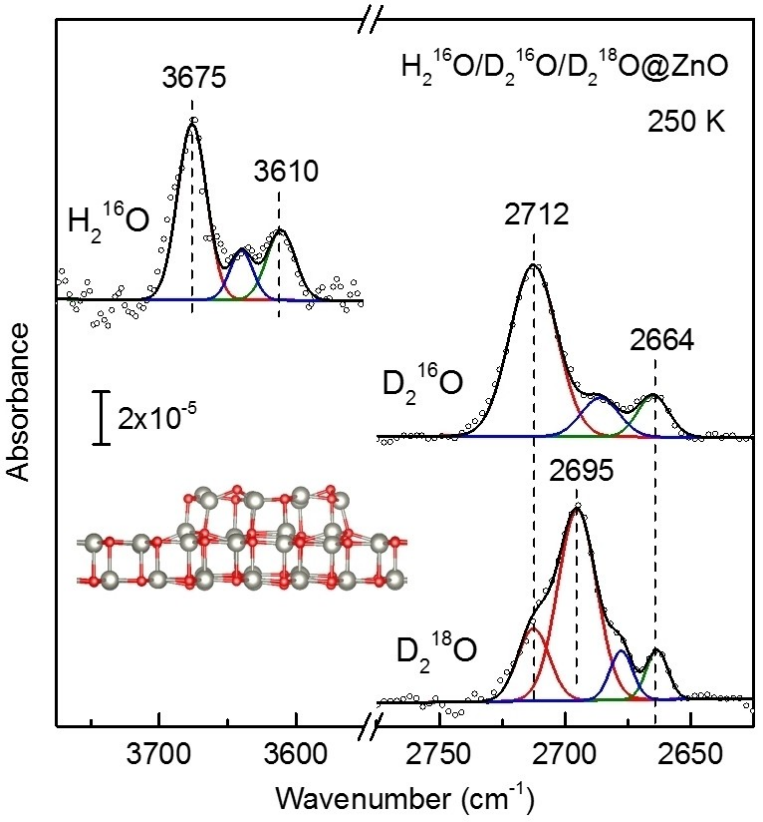
Comparison of IRRAS spectra recorded after exposing ZnO bilayer‐supported ZnO clusters to 1 L of H_2_^16^O, D_2_^16^O and D_2_^18^O at 250 K. The spectra were deconvoluted by fitting individual components with Gaussian curves.

The assignment of various hydroxyl species is aided by isotopic substitution measurements with H_2_^16^O (Figure 6), in which two IR bands at 3675 and 3610 cm^−1^ show the expected isotope shift of 1.36 with respect to the corresponding ^16^OD bands. In order to unambiguously identify the two OD species, further experiments with isotopically labeled D_2_^18^O were performed. After D_2_^18^O adsorption on ZnO clusters (Figure 6), two OD vibrations were seen at 2695 and 2664 cm^−1^. Note that the weak signal at 2712 cm^−1^ is due to the existence of a trace amount of D_2_^16^O. The 2695 cm^−1^ band red‐shifts by 17 cm^−1^ with respect to the ^16^OD vibration at 2712 cm^−1^, whereas another one at 2664 cm^−1^ does not change in frequency. These observations clearly prove that the IR band at 2664 cm^−1^ is attributed to ^16^OD species formed via D transfer from D_2_^18^O to the surface lattice oxygen (^16^O_s_D), while the 2695 cm^−1^ band originates from ^18^OD groups created by D_2_^18^O dissociation (^18^O_w_D) (see Figure 6). This is quite different from the case of partial dissociation of water on the wurtzite‐type ZnO(10$\bar{1}$ 0) single crystal surface.^[32]^ In addition, a minor hydroxyl species was resolved in the deconvoluted spectra (the blue one in Figure 6). This hydroxyl species could be formed via the dissociative adsorption of water on different Zn sites exposed by ZnO clusters, as supported by DFT simulations.

The full dissociation of water on ZnO clusters formed on graphitic ZnO bilayer at 250 K is further confirmed by the corresponding grazing‐emission XPS data of O 1s core level (Figure S5). In addition to the lattice O‐related signal at 530.1 eV,^[41]^ the second component at 531.7 eV is characteristic for the presence of hydroxyl groups.[^42^, ^43^, ^44^, ^45^] Furthermore, no D_2_O‐related O 1s peak at ∼533 eV was detected, indicating the full dissociation of water on ZnO clusters, in excellent agreement with the IRRAS observation.

Overall, the combined IRRAS and XPS results demonstrate that the small ZnO clusters formed on well‐ordered ZnO bilayers account for the high reactivity of Ag(111)‐supported ZnO thin films for water dissociation. This could be attributed to the presence of under‐coordinated Zn and O atoms exposed at the edges of ZnO clusters, as corroborated by DFT calculations.

In order to construct DFT models of the ZnO clusters we started from the STM observation that the ZnO islands grow with a triangular shape.[^22^, ^25^] We focused on the edges of these islands. There are two different types of (regular) triangles possible, and the edges consist either of a) three‐coordinated oxygen, O_3c_, and four‐coordinated zinc, Zn_4c_, atoms or b) four‐coordinated oxygen, O_4c_, and three‐coordinated zinc, Zn_3c_, atoms. Triangular islands are difficult to model since they are non‐stoichiometric (see Ref. [46] for an in‐depth analysis on the structure of different islands in ZnO/Ag(111)). We used two stoichiometric islands to model the experimental shape (see Figures S6, S7 and Supporting Information).

First, we considered molecular and dissociated water adsorption on top of the islands where no under‐coordinated atoms are involved in the binding process. The small rumpling in the third ZnO layer does not significantly affect the adsorption energy (−0.35 eV on bilayer, −0.42 eV on top of the island) as well as the geometry of molecular adsorption. In this respect, also the vibrational modes are not expected to change. Dissociative adsorption on neighboring atoms leads to a spontaneous recombination; dissociated water can be stabilized only if the fragments are moved away from each other. This adsorption mode on top of the island has an adsorption energy of −0.38 eV (+0.10 eV on the bilayer).

**Figure 7:**
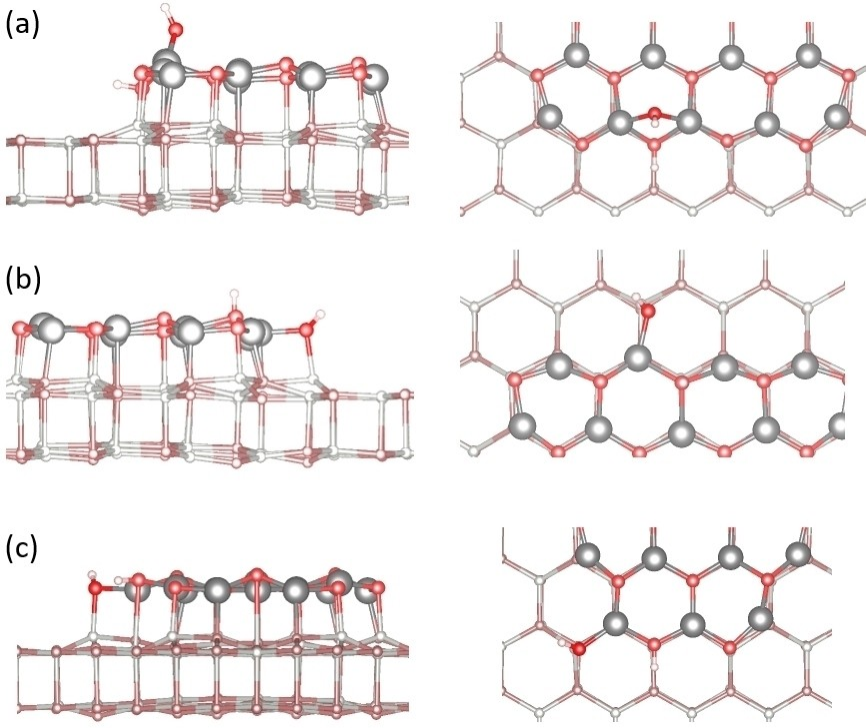
DFT‐optimized structures of dissociated water (a) on the edge with four coordinated Zinc atoms (Zn_4c_) and three coordinated oxygen atoms (O_3c_); (b) on the edge with three coordinated Zinc atoms (Zn_3c_) and four coordinated oxygen atoms (O_4c_); (c) on the tip of an island with Zn_3c_ atoms and O_3c_ atoms. Left panel: top view, Right panel: side view. For clarity, the atoms in the bilayer are displayed smaller and with less saturated colors. Color code: O (red), Zn (gray), H (white).

For the adsorption on the edges of the ZnO islands we considered the two types of edges and we placed an OD and D fragment on neighboring Zn and O atoms (see Figure 7 and Table 2). When the OD is adsorbed on top of Zn_4c_ it forms a bridging species (Figure 7a). This behavior is not found if the OD fragment is adsorbed on the edge with Zn_3c_ (Figure 7b). The adsorption energies for both modes are comparable, about −1.1 eV, and a small difference is found in the ν(O_surf_‐D), roughly 15 cm^−1^, Table 2. The adsorption energy is considerably higher than the molecular adsorption of a water monomer, dimer, and superstructure on the ZnO bilayer and of the dissociated species on top of the island. The higher adsorption energy is consistent with the high thermal stability of the experimental signals (see Figure 5).

**Table 2:** Computed adsorption energies and vibrational frequencies for the adsorption of dissociated water on the edges of the unsupported islands.

Adsorption site	E_ads_ [eV]	ν (Zn_surf_−O−D) [cm^−1^]	ν (O_surf_−D) [cm^−1^]
(Zn_4c_)_2_−OD and O_3c_−D	−1.12	2754.6 (−48.5)	2702.6 (−100.5)
Zn_3c_−OD and O_4c_−D	−1.15	2752.6 (−50.4)	2688.7 (−114.4)

At the tip of the ZnO triangular islands one can see the presence of Zn_3c_ and O_3c_ atoms. To simulate this case, we used a smaller island model (see Figure 7c). Adsorption of D and OD fragments on both under‐coordinated atoms leads to an adsorption energy of −1.79 eV, considerably higher (in absolute terms) than what found for the edges, −1.15 eV. Although these sites are more reactive, these species will be a minority because there are only three tips per ideal triangular island, hence a small number of Zn_3c_ and O_3c_ sites. We repeated these adsorption modes on the Ag supported ZnO films, but once more we did not find a considerable effect of the support on the chemistry of the system.

Overall, there is a good agreement between experiment and theory in both frequencies and binding energies of various hydroxyl groups formed by water dissociation at under‐coordinated Zn and O atoms exposed by supported ZnO clusters at the edges and corners. Furthermore, the IR spectra show that the O_s_D‐related vibration at 2664 cm^−1^ is much less intense than the 2712 cm^−1^ band (see Figure 5). This finding corresponds more likely to water dissociation on the edges exposing Zn_4c_ and O_3c_ atoms, as shown in Figure 7a. In this case, the Zn_4c_‐OD group adopts a nearly perpendicular geometry whereas the O_s_D species is oriented more parallel to the surface.

### 2.3 Coadsorption of D_2_O and CO on ZnO Bilayer‐Supported ZnO Clusters

**Figure 8:**
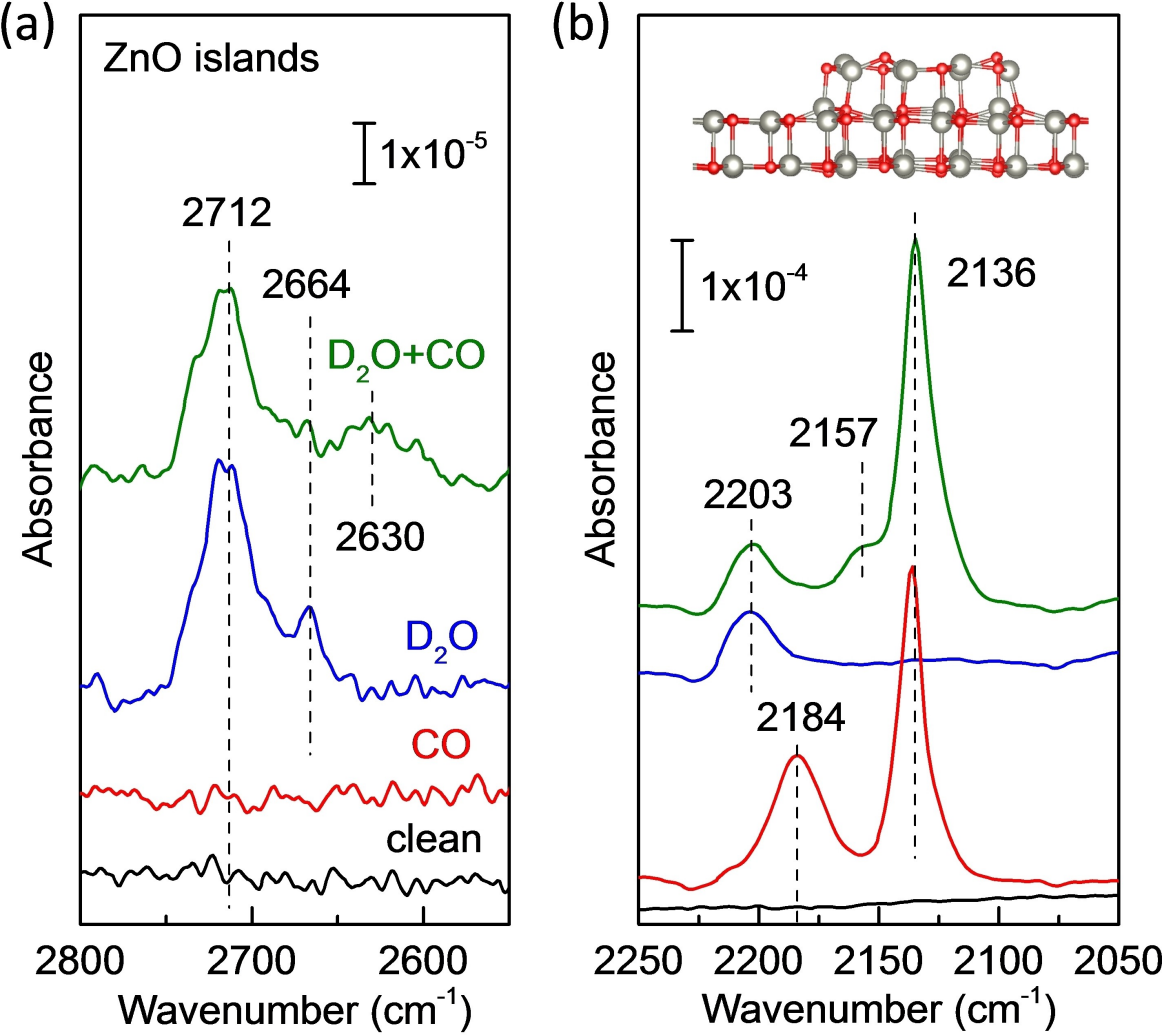
IRRA spectra obtained by exposing ZnO clusters supported on ZnO bilayer to 1 L CO at 56 K, 1 L D_2_O at 220 K, and first 1 L D_2_O at 220 K and then 1 L CO at 56 K, respectively. (a) Region of OD vibrations; (b) region of CO vibrations.

The high reactivity of ZnO clusters is further supported by the co‐adsorption experiments with CO as a probe molecule (Figure 8a and b). Two CO vibrational bands at 2136 and 2184 cm^−1^ are observed after CO adsorption at 56 K (Figure 8b), which are characteristic for the coexistence of graphitic ZnO bilayer and thicker ZnO islands, as discussed below. The weak and broad feature at 2184 cm^−1^ compared to CO adsorbed on well‐ordered ZnO single crystals indicates the formation of irregular, small ZnO clusters. The CO bands disappear completely after heating to 220 K. Upon a subsequent exposure of 1 L D_2_O at 220 K the spectrum (Figure 8a) shows clearly two individual hydroxyl groups at 2712 (^16^O_w_D) and 2664 cm^−1^ (^16^O_s_D) as well as the Zn‐OD phonon vibration at 2203 cm^−1^. Given that the graphitic ZnO bilayer is inactive for water dissociation, after D_2_O pretreatment at 220 K only the ZnO clusters are occupied by reacted OD species whereas the ZnO bilayer remains uncovered and is available for CO adsorption. Indeed, after readsorption of CO at 56 K (see Figure 8b), the ZnO bilayer‐related CO vibration at 2136 cm^−1^ does not change in intensity whereas the 2184 cm^−1^ band vanishes completely.

**Figure 9:**
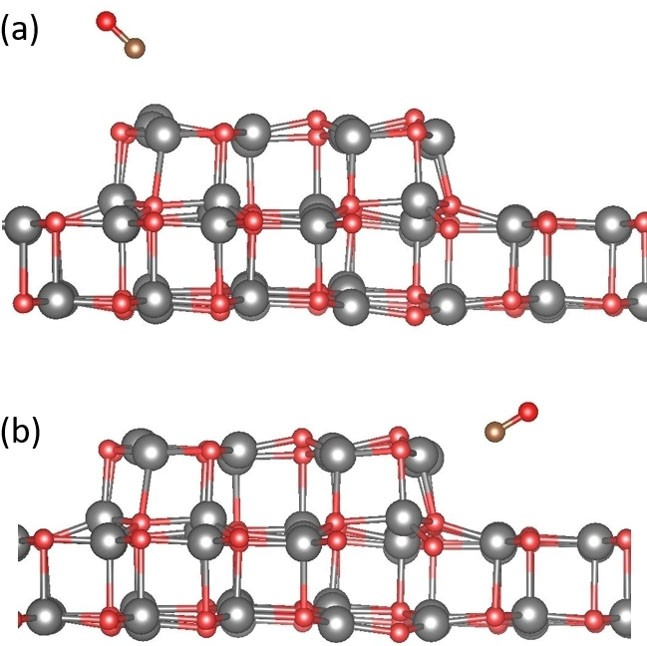
DFT‐optimized structures of CO binding to (a) a four‐coordinated Zn atom (Zn_4c_) and (b) a three‐coordinated Zn atom (Zn_3c_) at the edges of an ZnO island. Color code: O (red), Zn (gray), C (brown).

To further analyze the broad CO band at 2184 cm^−1^, we modeled the adsorption of CO on top as well as on the edges of islands. These are characterized by either Zn_4c_ and O_3c_ or Zn_3c_ and O_4c_ sites, Figure 9 and Table 3.

**Table 3:** Computed adsorption energies and vibrational frequencies of CO adsorbed at the ZnO islands.

Region	site	E_ads_ [eV]	ν (CO) [cm^−1^]
Edge	Zn_4c_	−0.29	2113.0 (−12.5)
Edge	Zn_3c_	−0.47	2159.0 (+32.5)
Terrace	Zn(1)	−0.23	2110.0 (−15.5)
Terrace	Zn(2)	−0.34	2161.2 (+35.7)
Terrace	Zn(3)	−0.30	2153.2 (+27.8)

Both adsorption modes show a significant tilt of CO towards the surface, especially for the adsorption on Zn_3c_. The adsorption energy on Zn_4c_ is only 0.05 eV larger than on the bilayer. The vibrational frequency of CO on Zn_4c_ shows a red‐shift of −12.5 cm^−1^ while a blue‐shift of +9.0 cm^−1^ is found for the bilayer. On the Zn_3c_ edge sites the adsorption energy is −0.47 eV and the CO stretching mode shows a blue‐shift of +32.5 cm^−1^.

Next, we considered CO adsorption on top of the islands (Figure S8 and Table 3). Due to the limited size of the model, all these sites are close to the edges of the island. It is apparent that on the terraces of the islands, while the adsorption energies are similar, the vibrational shifts change as a function of the chemical environment and of tilt angle of the CO molecule which is tilted by about 25° for adsorption site 1, by 8° on site 2 and 18° on site 3. The heterogeneity of the sites reflects the small size of our models of the islands.

To model adsorption on top of bigger islands we used a new model formed by three Zn−O layers. Here CO adsorbs with an adsorption energy of −0.25 eV similar to that found on the bilayer (−0.21 eV). The CO vibrational mode experiences a blue shift of +20.8 cm^−1^ (+9.0 cm^−1^ on the bilayer). This shows that the adsorption of CO on top of thicker ZnO islands can result in a sizable blue shift in the CO stretching mode without the need to assume that a transition to the wurtzite structure has occurred. This is consistent with a recent theoretical analysis^[19]^ that showed that for free‐standing ZnO films, a flat graphite‐like morphology is preferred up to 10 layers, where a transition to wurtzite‐like structures occurs. On Cu(111), a slightly corrugated graphitic structure is obtained for ZnO films up to 4 layers. For thicker films, the rumpling becomes substantial and a phase transition to wurtzite takes place. This suggests that three‐layers thick ZnO islands are not yet sufficient to reach a transition to a wurtzite structure.

A closer analysis of the IRRAS data shows that CO adsorption on the hydroxylated ZnO clusters leads to a significant attenuation of the OD bands at 2712 and 2664 cm^−1^ (Figure 8a). Simultaneously, a broad IR feature at about 2630 cm^−1^ and a shoulder at 2157 cm^−1^ are unambiguously resolved. The 2157 cm^−1^ band is attributed to CO weakly bound to acidic OD groups,^[47]^ which should account for the red shift in frequency observed for the OD vibrations, as corroborated by DFT calculations (see below). Overall, these results provide evidence that the small ZnO clusters with under‐coordinated Zn and O atoms at edges and corners are responsible for the extremely high reactivity for water dissociation.

**Figure 10:**
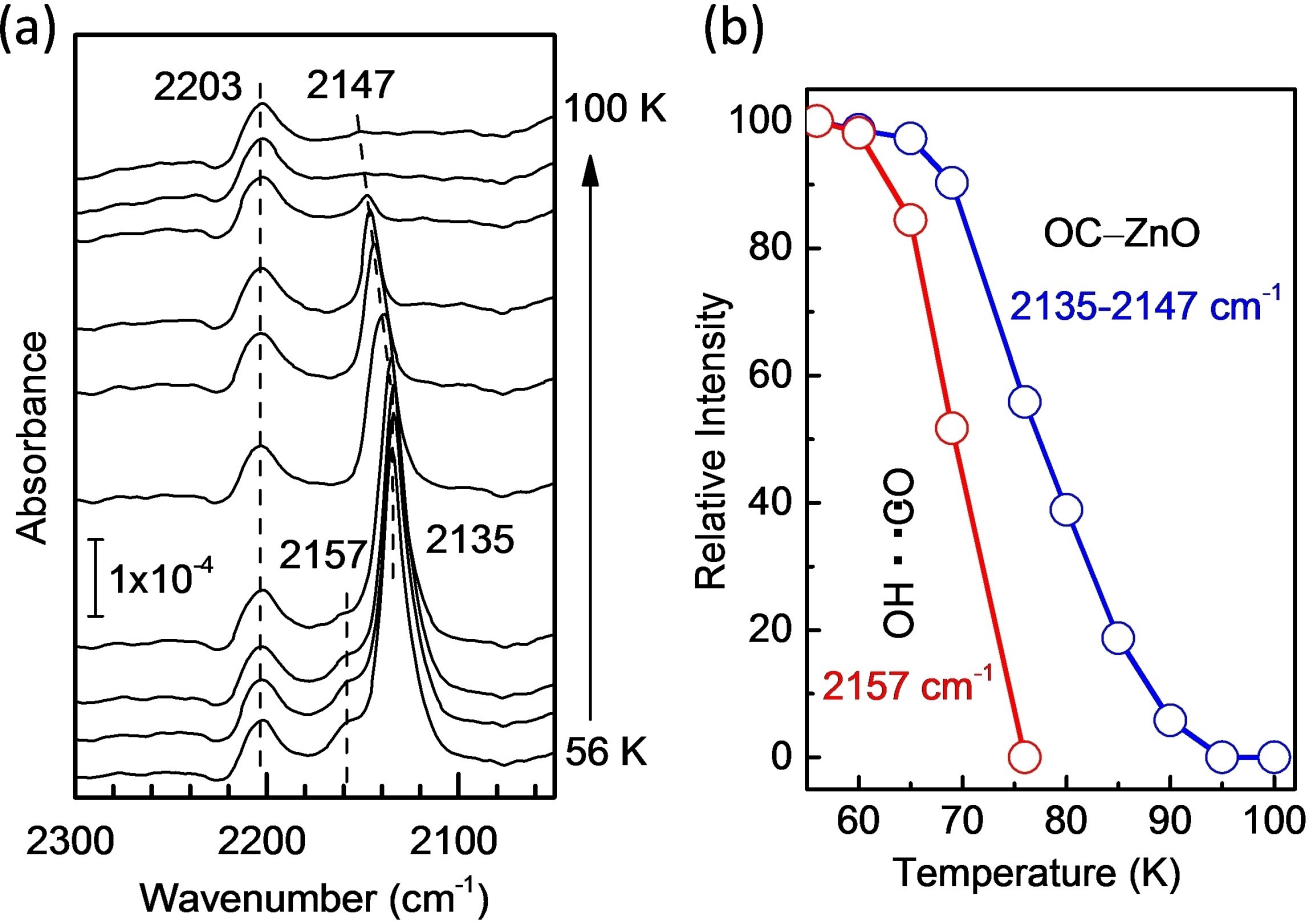
(a) Temperature‐dependent IRRAS spectra recorded after exposing ZnO bilayer supported ZnO clusters first to D_2_O at 220 K and then to 1 L CO at 56 K. The sample was heated gradually to 100 K. (b) Normalized intensity of the different bands as a function of sample temperature.

The temperature‐dependent IRRAS data, obtained for CO adsorption on D_2_O pre‐treated sample (see Figure 10a), allow gaining insight into the thermal stability of various CO species. Based on a quantitative analysis of the IR data (Figure 10b) and assuming a pre‐exponential factor of 10^13^ s^−1^, the binding energy of CO adsorbed on graphitic ZnO bilayer (2135–2147 cm^−1^) was determined to amount to 0.23 eV (22 kJ/mol). In addition, the shoulder at 2157 cm^−1^ disappears upon heating slightly to 75 K, revealing a very weak interaction of CO with hydroxyl groups via H‐bonding (OC ⋅ ⋅ ⋅ HO), as observed for CO adsorption on the hydroxylated O‐terminated ZnO(000$\bar{1}$ ) surface.^[48]^

Finally, in order to unambiguously identify the CO species bound to hydroxyl groups, additional experiments on the ZnO(10$\bar{1}$ 0) single‐crystal surface were performed. Figure S9 presents the corresponding IRRAS results recorded at 75 K, in which an intense band at 2157 cm^−1^ is clearly observed, affirming the vibration of CO species adsorbed on surface hydroxyl groups. Again, this band disappears completely at around 80 K, revealing that CO is weakly bound to hydroxyl groups. More importantly, the OD stretch vibrations show a CO‐induced red shift in frequency from 2711 to 2691 cm^−1^, in line with the observation on ZnO clusters.

**Figure 11:**
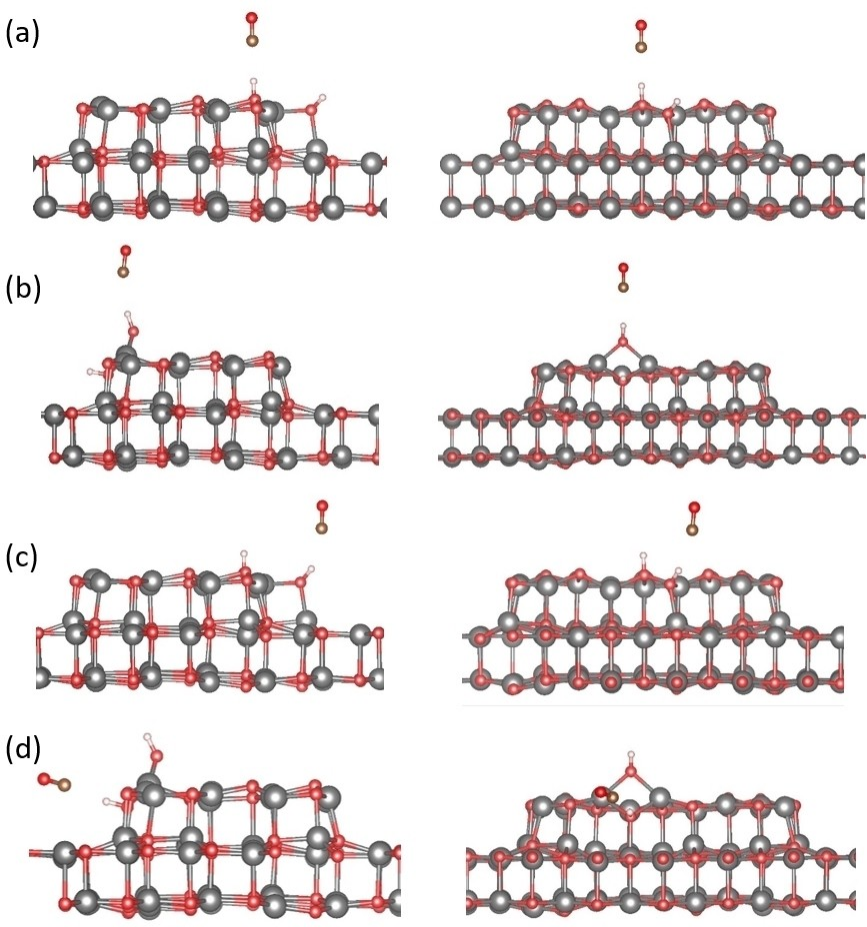
DFT‐optimized structures of CO co‐adsorbed to different OD groups formed via D_2_O dissociation on ZnO clusters, (a) isomer 1; (b) isomer 2; (c) isomer 3; (d) isomer 4. Color code: O (red), Zn (gray), C(brown), H (white).

To model CO and D_2_O co‐adsorption in DFT we chose as a starting point the dissociated D_2_O adsorbed at the edges of the island. Then we added a CO molecule on top of the OD and D fragments and carried out a vibrational analysis (Figure 11 and Table 4). The adsorption energies of CO on an OD group vary from weak bonding (isomers 2 and 3) to comparable strengths of the adsorption of CO on a surface Zn atom (isomers 1 and 4). The CO stretching frequency is either blue‐shifted (isomers 1–3) or does not change (isomer 4) while the OD stretching always experiences a strong red shift. Of course, the studied isomers represent only a small fraction of the possible structures. Nevertheless, the results are consistent with the experimental observations, and point to the formation of OH⋅⋅⋅CO surface complexes at the borders of the ZnO islands.

**Table 4:** Computed adsorption energies and vibrational frequencies of the co‐adsorbed CO molecule from DFT calculations. The CO frequency is compared to the gas‐phase value, the OD shift is referred to the OD group without adsorbed CO.

Adsorption type	Fragment	E_ads_ [eV] (of CO)	ν (CO) [cm^−1^]	ν (OD) [cm^−1^]
Isomer 1	D	−0.29	2164.8 (+39.3)	2505.4 (−183.3)
Isomer 2	OD	−0.13	2138.7 (+13.3)	2708.8 (−45.8)
Isomer 3	OD	−0.17	2145.7 (+20.2)	2684.5 (−68.2)
Isomer 4	D	−0.27	2125.2 (−0.3)	2648.4 (−54.1)

## 3 Conclusions

Taken together the experimental and theoretical results allow to draw a rather concise picture of the chemical reactivity of ZnO bilayers supported on Ag(111) substrates as well as of small ZnO clusters grown on top of these bilayers. In contrast to the bulk wurtzite ZnO(10$\bar{1}$ 0) surface, where the strong interaction leads to a partial dissociation of water molecules,^[32]^ the interaction between the graphitic ZnO bilayers and water is rather weak. Both, experiment and theory suggest that water adsorbs in an associate fashion on the well‐ordered planar ZnO bilayers.

A rather different scenario is encountered when small clusters or islands of zinc oxide are formed on the surface of the rather inactive ZnO bilayers. The theoretical results reveal that the structure of the ZnO clusters is quite different from that of the flat ZnO bilayer. The computed frequencies and binding energies for different CO species are in good agreement with those observed in IRRAS. Importantly, the chemical activity of these ZnO clusters is much higher than that of the bilayers as demonstrated by the fact that after exposure to water at 250 K, hydroxyl vibrations are clearly observed in the experimental infrared data. The dissociation of water on ZnO clusters, which appears to occur with a small activation barrier, is confirmed by the DFT calculations. The ^16^OH/^16^OD/^18^OD species formed in this process were identified directly by the corresponding stretching frequencies but also by the red shift in frequency upon CO adsorption on these acidic OH groups via H‐bonding. The under‐coordinated O and Zn atoms at the edges and corners account for the significantly enhanced reactivity of small ZnO clusters formed on the graphitic ZnO bilayers.

## Experimental and Theoretical Section

### Experimental Methods

The *in situ* IRRAS experiments were performed in an advanced UHV apparatus, which combines a state‐of‐the‐art FTIR spectrometer (Bruker Vertex 80v) with several other surface‐sensitive techniques (XPS and LEED).^[49]^ The innovative design not only allows us to record IRRAS data at grazing incidence on model oxide catalysts, but also enables transmission IR experiments on oxide powders supported on an inert metal mesh. This apparatus has been optimized for sensitivity and allows to reliably detect absorbances as low as 1×10^−5^, a prerequisite for detecting the vibrational signatures of adsorbates on oxide single‐crystal substrates.[^49^, ^50^]

The clean AgZn(111) single‐crystal surface (Ag/Zn ratio 9 : 1) was prepared by cycles of Ar^+^ sputtering (1.5 kV, 10 mA, 1×10^−6^ mbar) at 300 K and subsequent annealing at 420 K. The cleanliness and oxidation states of the sample were monitored by XPS equipped with a Scienta R4000 electron energy analyzer. The ZnO thin layers grown on the AgZn(111) substrate were prepared by oxidation of the clean AgZn(111) surface in O_2_ atmosphere (1×10^−5^ mbar) at different temperatures for different periods of time. The thickness of ZnO adlayers was determined based on a quantitative analysis of the Zn 2p_3/2_ and Ag 3d_5/2_ XPS signals (for detailed analysis please see Ref. [28]).

Exposure to CO at 60 K (cooling with liquid helium) as well as water (H_2_^16^O, D_2_^16^O and D_2_^18^O) at 110 K (cooling with liquid nitrogen) was carried out by backfilling the IR chamber using a leak‐valve‐based directional doser connected to a tube (2 mm in diameter) that terminated 3 cm from the sample surface. The purity was 99.0 % for D in D_2_O. Additional purification of H_2_O and D_2_O was achieved by repeated cycles of freezing, pumping and thawing. IRRAS data were accumulated by recording 1024 scans with a resolution of 4 cm^−1^. Prior to each exposure, a spectrum of a clean sample was recorded as a background reference. Exposures are given in units of Langmuir (L) (1 L=1.33×10^−6^ mbar s). The binding energy of adsorbed CO species was estimated based on temperature‐dependent IRRAS data using the Redhead equation^[51]^ assuming a pre‐exponential factor of 10^13^ s^−1^.

## Computational Details

We carried out the DFT calculations using the Vienna Ab‐Initio Simulation Package (VASP).[^52^, ^53^] The projector augmented wave (PAW) method[^54^, ^55^] was used to describe the interactions between the core electrons and the nuclei. We applied the Perdew‐Bruke‐Enzerhof (PBE) exchange‐correlation functional.^[56]^ The valence electrons (Ag(5 s, 4d), Zn(3d, 4p), C(2 s, 2p), O(2 s, 2p), H(1 s)) were treated explicitly. We relaxed all structures until the residual forces on each atom were below 0.02 eV/Å. We use a kinetic cutoff energy of 400 eV for the surface models. We kept a vacuum distance of at least 20 Å to reduce interaction between slabs.

The lattice parameter of fcc‐Ag has been relaxed with a kinetic energy cutoff of 600 eV. The result is a=4.079 Å, in good agreement with the experimental result, 4.0857 Å^[57]^ (0.16 % deviation). It has been shown that the ZnO bilayer in hexagonal boron nitride structure forms a superlattice, where a 7×7 bilayer of ZnO interacts with a 8×8 Ag(111) surface.^[22]^

To reduce computational cost, we considered an unsupported bilayer of ZnO, where the lattice parameters are scaled according to the results obtained for the same layer on a silver support. We also considered a supported model, where a bilayer of ZnO binds to four layers of the silver support, ZnO/Ag(111). However, previous studies showed that the metal support does not affect the surface chemistry significantly.^[27]^ In a related study of ZnO on Au(111) the adsorption energy of a water monomer changed by 0.4 kcal/mol by adding the metal support to the model.^[25]^

Optimizations on the big surface model (7×7 ZnO on 8×8 Ag(111)) were carried out on the Γ‐point; optimizations of the small surface model (3×3 ZnO) were done using a 2×2×1 k‐point grid. For the unsupported case we employed two different sizes for the bilayer model. We used a 3×3 model for the bilayer and a 7×7 supercell to model the third‐layer islands. Due to the 7×7 ZnO/8×8 Ag(111) superlattice we could only use the bigger model for the supported case.

All calculated frequency shifts are given with respect to gas phase molecules. For D_2_O, unless otherwise stated, we used the asymmetric stretching vibration ν_3,calc_(gas phase)=2803.0 cm^−1^ and for CO ν_CO,calc_=2125.5 cm^−1^. The frequencies have not been scaled. A negative adsorption energy indicates an exothermic process.

## Conflict of interest

The authors declare no conflict of interest.

## Supporting information

As a service to our authors and readers, this journal provides supporting information supplied by the authors. Such materials are peer reviewed and may be re‐organized for online delivery, but are not copy‐edited or typeset. Technical support issues arising from supporting information (other than missing files) should be addressed to the authors.

Supplementary

## References

[1] A. Trovarelli, P. Fornasiero, *Catalysis by Ceria and Related Materials*, Imperial College Press, London, **2013**.

[2] M. Behrens , F. Studt , I. Kasatkin , S. Kuhl , M. Havecker , F. Abild-Pedersen , S. Zander , F. Girgsdies , P. Kurr , B.-L. Kniep , M. Tovar , R. W. Fischer , J. K. Norskov , R. Schlogl , Science 2012, 336, 893–897. 22517324 10.1126/science.1219831

[3] C. Wöll , Prog. Surf. Sci. 2007, 82, 55–120.

[4] U. Diebold , Surf. Sci. Rep. 2003, 48, 53–229.

[5] P. A. Thiel , T. E. Madey , Surf. Sci. Rep. 1987, 7, 211–385.

[6] M. A. Henderson , Surf. Sci. Rep. 2002, 46, 1–308.

[7] R. Mu , Z. J. Zhao , Z. Dohnálek , J. Gong , Chem. Soc. Rev. 2017, 46, 1785–1806. 28180223 10.1039/c6cs00864j

[8] T. Hisatomi , J. Kubota , K. Domen , Chem. Soc. Rev. 2014, 43, 7520–7535. 24413305 10.1039/c3cs60378d

[9] T. Lunkenbein , J. Schumann , M. Behrens , M. R. Schlögl , M. G. Willinger , Angew. Chem. Int. Ed. 2015 54, 4544–4548. 10.1002/anie.201411581 25683230

[10] F. Studt , M. Behrens , E. L. Kunkes , N. Thomas , S. Zander , A. Tarasov , J. Schumann , E. Frei , J. B. Varley , F. Abild-Pedersen , J. K. Nørskov , R. Schlögl , ChemCatChem 2015, 7, 1105–1111.

[11] C. Ratnasamy , J. Wagner , Catal. Rev. Sci. Eng. 2009, 51, 325–440.

[12] S. Kuld , M. Thorhauge , H. Falsig , C. F. Elkjær , S. Helveg , I. Chorkendorff , J. Sehested , Science 2016, 352, 969–974. 27199425 10.1126/science.aaf0718

[13] S. Kattel , P. J. Ramírez , J. G. Chen , J. A. Rodriguez , P. Liu , Science 2017 355, 1296–1299. 28336665 10.1126/science.aal3573

[14] J. L. Santos , T. R. Reina , S. Ivanova , M. A. Centeno , J. A. Odriozola , Appl. Catal. B 2017, 201,310–317.

[15] Z. Boukha , J. L. Ayastuy , J. R. González-Velasco , M. A. Gutiérrez-Ortiz , Appl. Catal. A 2018, 566, 1–14.

[16] S. Wang , G. Liu , L. Wang , Chem. Rev. 2019, 119, 5192–5247. 30875200 10.1021/acs.chemrev.8b00584

[17] V. Schott , H. Oberhofer , A. Birkner , M. Xu , Y. Wang , M. Muhler , K. Reuter , C. Wöll , Angew. Chem. Int. Ed. 2013 52, 11925–11929. 10.1002/anie.201302315 24105810

[18] Q. Pan , B. H. Liu , M. E. McBriarty , Y. Martynova , I. M. N. Groot , S. Wang , M. J. Bedzyk , S. Shaikhutdinov , H. J. Freund , Catal. Lett. 2014, 144, 648–655.

[19] H. V. Thang , S. Tosoni , G. Pacchioni , Appl. Surf. Sci. 2019, 483, 133–139.

[20] F. Claeyssens , C. L. Freeman , N. L. Allan , Y. Sun , M. N. R. Ashfold , J. H. Harding , J. Mater. Chem. 2005 15, 139–148.

[21] C. L. Freeman , F. Claeyssens , N. L. Allan , J. H. Harding , Phys. Rev. Lett. 2006, 96, 066102. 16606013 10.1103/PhysRevLett.96.066102

[22] C. Tusche , H. L. Meyerheim , J. Kirschner , Phys. Rev. Lett. 2007, 99, 026102. 17678236 10.1103/PhysRevLett.99.026102

[23] A. Shiotari , B.-H. Liu , S. Jaekel , L. Grill , S. Shaikhutdinov , H.-J. Freund , M. Wolf , T. Kumagai , J. Phys. Chem. C 2014, 118, 27428–27435.

[24] B. H. Liu , J. A. Boscoboinik , Y. Cui , S. Shaikhutdinov , H. J. Freund , J. Phys. Chem. C 2015, 119, 7842–7847.

[25] X. Deng , D. C. Sorescu , J. Lee , J. Phys. Chem. C 2016, 120, 8157–8166.

[26] H. Q. Ta , L. Zhao , D. Pohl , J. Pang , B. Trzebicka , B. Rellinghaus , D. Pribat , T. Gemming , Z. Liu , A. Bachmatiuk , M. H. Rümmeli , Crystals. 2016 6, 100.

[27] S. Tosoni , C. Li , C. P. Schlexer , G. Pacchioni , J. Phys. Chem. C 2017, 121, 27453–27461.

[28] M. Andersen , X. Yu , M. Kick , Y. Wang , C. Wöll , K. Reuter , J. Phys. Chem. C 2018, 122, 4963–4971.

[29] Y. Wang , X. Xia , A. Urban , H. Qiu , J. Strunk , B. Meyer , M. Muhler , C. Wöll , Angew. Chem. Int. Ed. 2007 46, 7315–7318. 10.1002/anie.200702815 17768757

[30] H. Noei , C. Wöll , M. Muhler , Y. Wang , Appl. Catal. A 2011, 391, 31–35.

[31] M. Buchholz , X. Yu , C. Yang , S. Heißler , A. Nefedov , Y. Wang , C. Wöll , Surf. Sci. 2016, 652, 247–252.

[32] X. Yu , P. Schwarz , A. Nefedov , B. Meyer , Y. Wang , C. Wöll , Angew. Chem. Int. Ed. 2019, 58, 17751–17757. 10.1002/anie.201910191 PMC6899783 31637780

[33] M. Schiek , K. Al-Shamery , M. Kunat , F. Traeger , C. Wöll , Phys. Chem. Chem. Phys. 2006, 8, 1505–1502. 16633634 10.1039/b515418a

[34] B. Meyer , D. Marx , O. Dulub , U. Diebold , M. Kunat , D. Langenberg , C. Wöll , Angew. Chem. Int. Ed. 2004, 43, 6642–6645. 10.1002/anie.200461696 15593168

[35] Y. Wang , M. Muhler , C. Wöll , Phys. Chem. Chem. Phys. 2006, 8, 1521–1524. 16633636 10.1039/b515489h

[36] H. Qiu , B. Meyer , Y. Wang , C. Wöll , Phys. Rev. Lett. 2008, 101, 236401. 19113570 10.1103/PhysRevLett.101.236401

[37] R. G. Greenler , D. R. Snider , D. Witt , R. S. Sorbello , Surf. Sci. 1982, 118, 415–428.

[38] R. Fuchs , K. L. Kliewer , Phys. Rev. 1965, 140, A2076–A2088.

[39] Y. Wang , R. Kováčik , B. Meyer , K. Kotsis , D. Stodt , V. Staemmler , H. Qiu , F. Traeger , D. Langenberg , M. Muhler , C. Wöll , Angew. Chem. Int. Ed. 2007, 46, 5624–5627; 10.1002/anie.200700564 17579889

[40] Y. Wang , Z. Phys. Chem. 2008, 222, 927–964.

[41] K. Kotsis , V. Staemmler , Phys. Chem. Chem. Phys. 2006, 8, 1490–1498. 16633632 10.1039/b515699h

[42] M. A. Haija , S. Guimond , A. Uhl , H. Kuhlenbeck , H. J. Freund , Surf. Sci. 2006, 600, 1040–1047.

[43] M. Chen , X. Wang , Y. H. Yu , Z. L. Pei , X. D. Bai , C. Sun , R. F. Huang , L. S. Wen , Appl. Surf. Sci. 2000, 158, 134–140.

[44] Y. Jeong , C. Bae , D. Kim , K. Song , K. Woo , H. Shin , G. Cao , J. Moon , ACS Appl. Mater. Interfaces 2010, 2, 611–615. 20356256 10.1021/am900787k

[45] M. S. Abdel-Wahab , A. Jilani , I. S. Yahia , A. A. Al-Ghamdi , Superlattices Microstruct. 2016, 94, 108–118.

[46] I. Demiroglu , S. T. Bromley , J. Phys. Condens. Matter 2016, 28, 224007. 26979335 10.1088/0953-8984/28/22/224007

[47] K. I. Hadjiivanov , G. N. Vayssilov , Adv. Catal. 2002, 47, 307–511.

[48] V. Staemmler , K. Fink , B. Meyer , D. Marx , M. Kunat , S. Gil Girol , U. Burghaus , C. Wöll , S. Phys. Rev. Lett. 2003, 90, 106102. 10.1103/PhysRevLett.90.106102 12689012

[49] Y. Wang , C. Wöll , Chem. Soc. Rev. 2017, 46, 1875–1932. 28221385 10.1039/c6cs00914j

[50] C. Wöll , ACS Catal. 2020 10, 168–176.

[51] P. A. Redhead , Vacuum 1962 12, 203–211.

[52] G. Kresse , J. Furthmüller , Comput. Mater. Sci. 1996 6, 15–50.

[53] G. Kresse , J. Furthmüller , Phys. Rev. B 1996, 54, 11169. 10.1103/physrevb.54.11169 9984901

[54] P. E. Blöchl , Phys. Rev. B 1994, 50, 17953. 10.1103/physrevb.50.17953 9976227

[55] D. Joubert , Phys. Rev. B 1999, 59, 1758–1775.

[56] J. P. Perdew , K. Burke , M. Ernzerhof , Phys. Rev. Lett. 1996, 77, 3865. 10062328 10.1103/PhysRevLett.77.3865

[57] *CRC Handbook of Chemistry and Physics*, 89th ed.; CRC Press, **2008**.

