# Supplementary material for: Chemical Reactivity of Supported ZnO Clusters: Undercoordinated Zinc and Oxygen Atoms as Active Sites
Source: Chemphyschem. 2020 Nov 13;21(23):2553–64. doi: 10.1002/cphc.202000747 (PMC7756222; doi:10.1002/cphc.202000747)
Supplement: Supplementary file 1 — Supplementary [file CPHC-21-2553-s001.pdf]

ChemPhysChem

Supporting Information

Chemical Reactivity of Supported ZnO Clusters: Undercoordinated Zinc and Oxygen Atoms as Active Sites

Xiaojuan Yu[+], Jannik P. Roth[+], Junjun Wang, Eric Sauter, Alexei Nefedov, Stefan Heißler, Gianfranco Pacchioni,* Yuemin Wang,* and Christof Wöll*

1. Structural evolution of on Ag(111)-supported ultrathin ZnO adlayers monitored by IRRAS

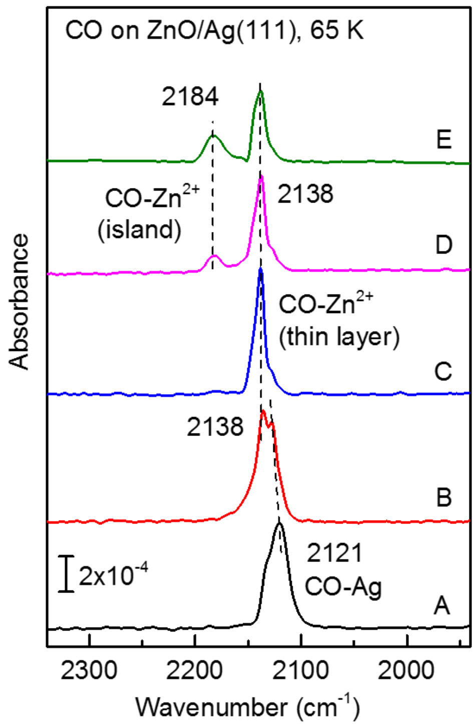

Figure S1. IRRAS data recorded after CO adsorption on the pristine and oxidized AgZn(111) surfaces at 65 K. The ZnO adlayers were prepared by oxidizing the (A) AgZn(111) surface in 1 x 10^{-5} mbar O_2 atmosphere at 500 K for (B) 10 min, and at 600 K for (C) 10 min, (D) 20 min, (E) 40 min. Adapted with permission from [1], Copyright 2018 American Chemical Society.

2. Structure of bilayer models

Optimizing the free-standing bilayer (with lattice parameters fixed to those of the Ag support) in the hexagonal boron nitride structure leads to a completely flat bilayer. This agrees with previous theoretical results[2]. To model the supported bilayer we used four layers of Ag atoms, while keeping the lowest layer fixed to its bulk positions. The optimization leads to flat bilayer on top of the Ag support. The silver atoms show a negligible movement away from the bulk positions. The interlayer distance is 2.66 Å, and there is a small charge transfer of 1.25 electrons from the Ag support to the ZnO bilayer (0.005 e^- per Ag atom, -0.013 e^- per ZnO formula unit) in good agreement with previous theoretical studies[2].

Upon adsorbing and removing adsorbates on this system, we found that this is not the only stable configuration for a bilayer on top of the silver support. We found a slightly perturbed surface more stable: here some zinc atoms of the lower layer move by ~0.5 Å towards the silver support, while the oxygen atom on top of the zinc atom moves up by about ~0.5 Å. We found that having about five of these zinc and oxygen pairs moving up and down stabilizes the surface by roughly 0.4 eV. Compared to the overall size of the system it is an almost negligible energy gain, but since the adsorption energy of water molecules are in the same order of magnitude it is important to take this structural change into account. For the free-standing bilayer such a distortion is not energetically favored. The charge transfer between support and ZnO bilayer is almost identical as in the other structure (1.48 electrons from silver support to ZnO bilayer). We could not find any previous report on this structure, neither experimental nor theoretical. It suggests a given flexibility of the supported ZnO bilayer which can accommodate slightly different structures with similar energies. We noticed, that the perturbance of the flat surface in the new supported model considerably reduces the dipole of the system. Since we are using a dipole correction, we tentatively suggest that the stabilization is due to an overcorrection of the dipole correction.

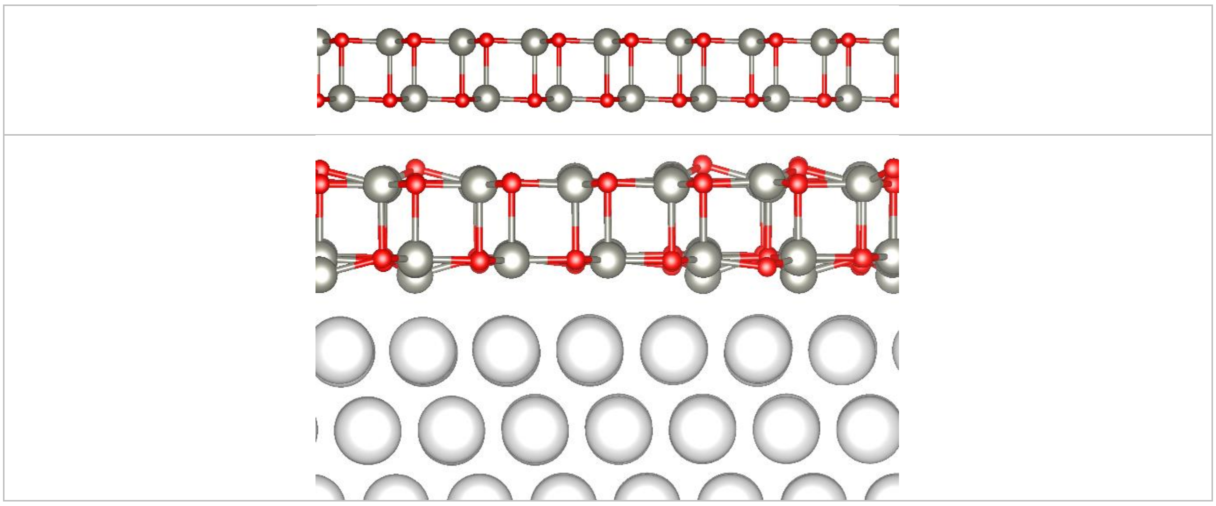

Figure S2. top: unsupported flat film, bottom: supported slightly perturbed film. Color code: Ag (light gray), O (red), Zn (gray).

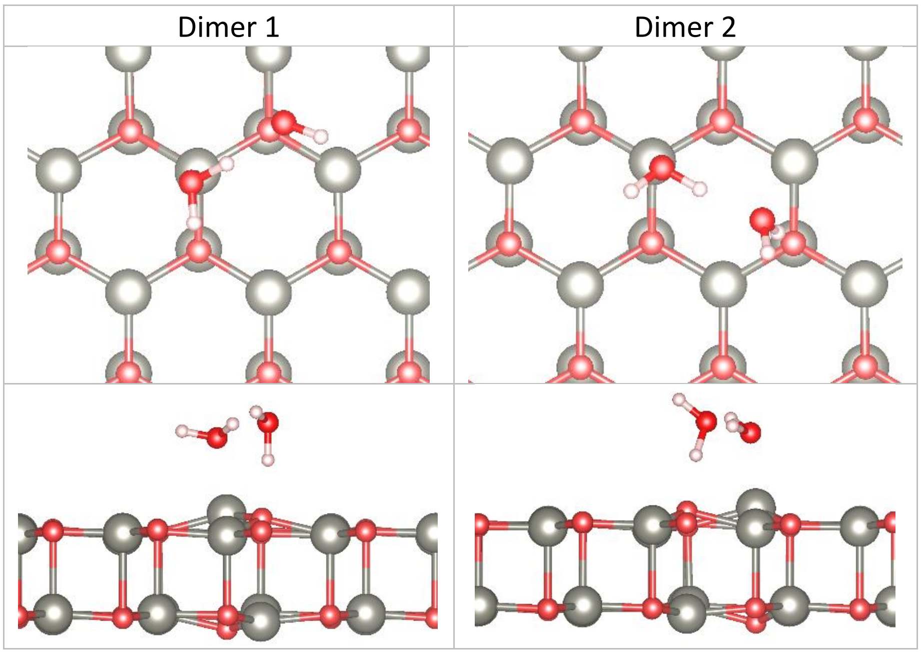

Figure S3. Top and side view for the discussed dimer structures. Color code O (red), Zn (gray), H (white).

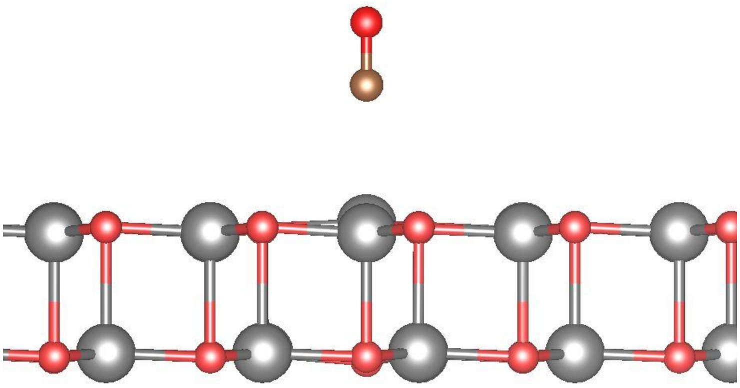

Figure S4. Adsorption configuration of CO on the intact bilayer. Color code: O (red), Zn (gray), C (brown).

3. D_2O adsorption on ZnO-bilayer-supported ZnO clusters characterized by XPS

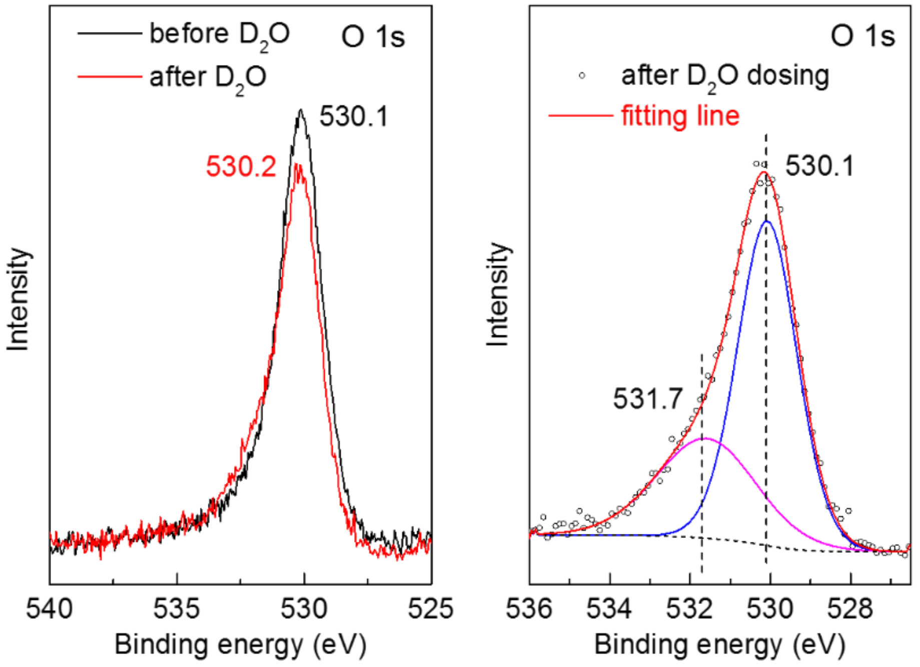

Figure S5. (a) Grazing-emission O 1s spectra recorded before (black curve) and after (red curve) 1 L D_2O adsorption on ZnO bilayer-supported ZnO clusters at 250 K; (b) the deconvoluted O 1s spectrum after water adsorption.

4. Structure of ZnO islands

The hexagonal island, Figure S6, shows a small structural change compared to the perfectly flat bilayer. In the unsupported model, the Zn atoms at the edge move inward by about 0.5 Å while the other atoms at the edge show no significant movement in the plane parallel to the surface. This behavior is in good agreement with results reported in the literature, see ref [3]. The O_{4c} atoms of the third layer move upwards (away from the surface) by about 0.5 Å while the Zn_{4c} atoms only move up by about 0.3 Å. The third layer is thereby no longer flat but shows a small rumpling.

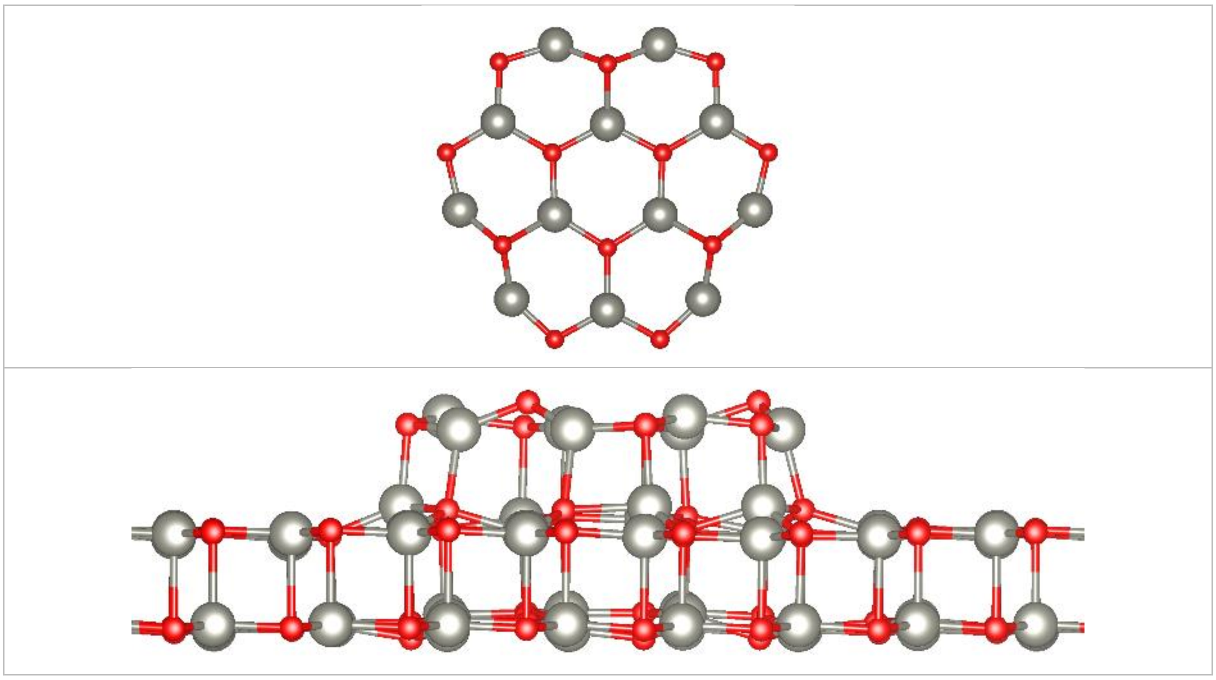

Figure S6. Top and side view of the hexagonal island. In the top view the bilayer below is not shown for clarity reasons. Color code: O (red), Zn (gray).

Going to the rectangular island, Figure S7, we see a similar behavior. For the unsupported case, the Zn_{3c} atoms move inward by about 0.5 Å while the O_{3c} atoms only move inward by about 0.2 Å in the plane parallel to the surface. At the O_{3c}/Zn_{4c} edge the Zn atoms move up by about 0.3 Å while the oxygen atoms move down by about 0.1 Å. At the O_{4c}/Zn_{3c} edge, the Zn atoms move down by about 0.2 Å while the O atoms move up by about 0.4 Å.

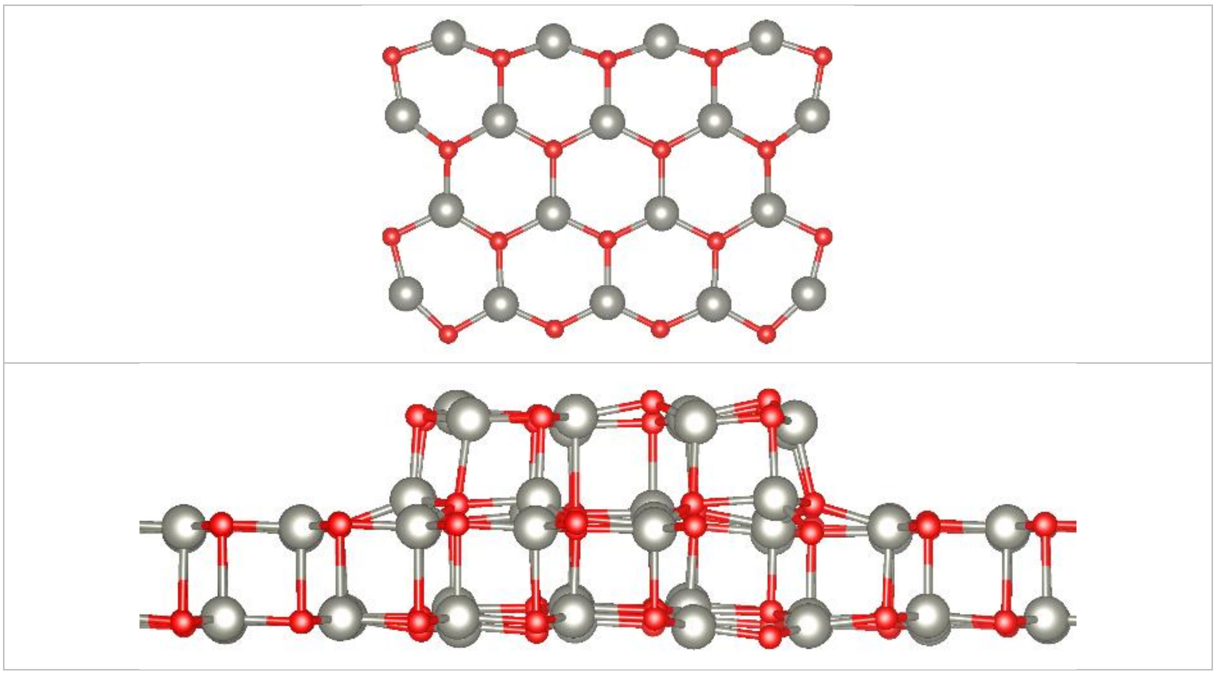

Figure S7. Top and side view of the rectangular island. In the top view the bilayer below is not shown for clarity reasons. Color code: O (red), Zn (gray).

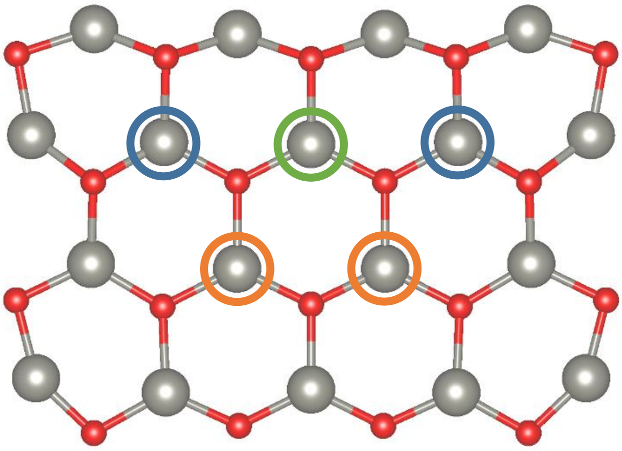

Figure S8. Different adsorption sites on top of the rectangular island. orange: adsorption site 1, green: adsorption site 2, blue: adsorption site 3. Color code: O (red), Zn (gray).

5. Adsorption of CO and D_2O on ZnO(10-10) single crystal surfaces monitored by IRRAS

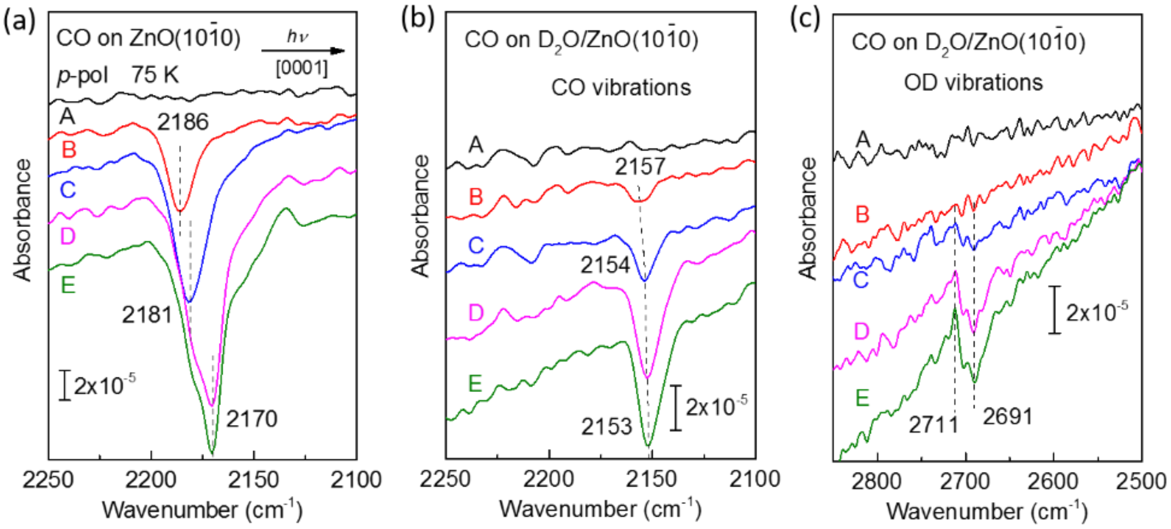

Figure S9. (a) IRRA spectra obtained by exposing the clean ZnO(10-10) single crystal surface to different doses of CO at 75 K. (b,c) IRRA spectra in the regions of CO (b) and OD vibrations (c) obtained by exposing the OD pre-covered ZnO(10-10) surface to various amount of CO at 75 K. All spectra were measured with *p*-polarized light incident along [0001] azimuth at 75 K.

References

[1] Andersen, M.; Yu, X.; Kick, M.; Wang, Y.; Wöll, C.; Reuter, K. Infrared Reflection–Absorption Spectroscopy and Density Functional Theory Investigations of Ultrathin ZnO Films Formed on Ag(111), *J. Phys. Chem. C* **2018**, 122, 4963-4971. https://doi.org/10.1021/acs.jpcc.8b00158.

[2] Tosoni, S.; Li, C.; Schlexer, P.; Pacchioni, G. CO Adsorption on Graphite-like ZnO Bilayers Supported on Cu(111), Ag(111), and Au(111) Surfaces. *J. Phys. Chem. C* **2017**. https://doi.org/10.1021/acs.jpcc.7b08781.

[3] Demiroglu, I.; Bromley, S. T. Evidence for Multi-Polymorphic Islands during Epitaxial Growth of ZnO on Ag(1 1 1). *J. Phys. Condens. Matter* **2016**. https://doi.org/10.1088/0953-8984/28/22/224007.
